# Prevalence of Pancreatic Steatosis and Its Associated Factors in Turkey: A Nation-Wide Multicenter Study

**DOI:** 10.5152/tjg.2024.23583

**Published:** 2024-03-01

**Authors:** Orhan Sezgin, Serkan Yaraş, Mehmet Cindoruk, Elmas Kasap, Hakan Ünal, Aydın Şeref Köksal, Abdullah Emre Yıldırım, Burak Özşeker, Nevin Oruç, Müjde Soytürk, Sabite Kaçar, Muhsin Kaya, Kader Irak, Yasemin Gökden, Deniz Öğütmen Koç, Osman Özdoğan, Engin Altıntaş, Nergiz Ekmen, Murat Saruç, Şencan Acar, Mahmut Polat, Sezgin Barutçu, Göksel Bengi, Volkan Gökbulut, Nalan Gülşen Ünal, Dilek Oğuz

**Affiliations:** 1 Department of Gastroenterology, Mersin University Faculty of Medicine, Mersin, Turkey; 2 Department of Gastroenterology, Gazi University Faculty of Medicine, Ankara, Turkey; 3 Department of Gastroenterology, Manisa Celal Bayar University Faculty of Medicine, Manisa, Turkey; 4 Department of Gastroenterology, Acıbadem Mehmet Ali Aydınlar University Faculty of Medicine, İstanbul, Turkey; 5 Department of Gastroenterology, Sakarya University Faculty of Medicine, Sakarya, Turkey; 6 Department of Gastroenterology, Memorial Bahçelievler Hospital, University of Gaziantep Faculty of Medicine, İstanbul, Turkey; 7 Department of Medicine, Johns Hopkins Hospital and Health System, Baltimore, Maryland, USA; 8 Department of Gastroenterology, Ege University Faculty of Medicine, İzmir, Turkey; 9 Department of Gastroenterology, Dokuz Eylül University Faculty of Medicine, İzmir, Turkey; 10 Department of Gastroenterology, Yüksek İhtisas Education and Research Hospital, Ankara, Turkey; 11 Department of Gastroenterology, Dicle University Faculty of Medicine, Diyarbakır, Turkey; 12 Department of Gastroenterology, İstanbul Kanuni Sultan Süleyman Training and Research Hospital, İstanbul, Turkey; 13 Department of Gastroenterology, University of Health Science Okmeydanı Training and Research Hospital, İstanbul, Turkey; 14 Department of Gastroenterology, Gaziosmanpaşa Taksim Education and Research Hospital, İstanbul, Turkey; 15 Department of Gastroenterology, Ministry of Health Amasya Sabuncuoğlu Şerefeddin Education and Research Hospital, Amasya, Turkey; 16 Department of Gastroenterology, Şanlıurfa Mehmet Akif İnan Education and Research Hospital, Şanlıurfa, Turkey; 17 Department of Gastroenterology, University of Gaziantep, Gaziantep, Turkey; 18 Department of Gastroenterology, Kırıkkale University Faculty of Medicine, Kırıkkale, Turkey

**Keywords:** Elastography, exocrine pancreatic insufficiency, lean steatosis, pancreas stiffness, pancreatic steatosis, ultrasonography

## Abstract

**Background/Aims:**

Pancreatic steatosis (PS) is a pathology associated with metabolic syndrome (MS), endocrin and exocrine disfunctions of the pancreas, and fatty liver. The data on the frequency of PS are very limited. We aimed to evaluate the frequency of PS detected by transabdominal ultrasonography (TAU) in gastroenterology clinics located in different geographical regions of Turkey and the factors associated with it.

**Materials and Methods:**

Volunteers were evaluated by TAU for PS and hepatosteatosis (HS), and its degree. Pancreatic stiffness was evaluated by ultrasonographic shear wave elastography (SWE). All demographic, physical, and biochemical parametres were measured.

**Results:**

A total of 1700 volunteers from 14 centers throughout Turkey were included in the study. Mean age was 48.03 ± 20.86 years (56.9% female). Prevalance of PS was detected in 68.9%. In the PS group, age, body mass index (BMI), waist circumference, systolic blood pressure, fasting blood glucose (FBG), lipid levels, insulin resistance, diabetes mellitus, hypertension, MS frequency, and pancreatic SWE score were increasing, and fecal elastase level was decreasing in correlation with the degree of PS. The frequency of HS was 55.5%. Hepatosteatosis [odds ratio (OR): 9.472], increased age (OR: 1.02), and BMI (OR: 1.089) were independent risk factors for the occurrence of PS. Lean-PS rate was 11.8%. The lean-PS group was predominantly female and younger than non-lean PS. Also it has lower blood pressure, FBG, liver enzymes, lipid levels, and HS rates.

**Conclusion:**

The frequency of PS was found 68.9% in Turkey. Its relationship was determined with age, BMI, HS, MS (and its components), pancreatic stiffness, and fecal elastase level.

**Main Points**

- Pancreatic steatosis (PS) and its stage is a valuable finding related to metabolic syndrome (MS), cardiovascular risk factors, and general well-being.
- Detection of PS is the first step to protect health and take precautions.
- Pancreatic steatosis and its stages can be quickly and simply detected by abdominal ultrasonography.
- There is no reliable information about its frequency. For this reason, we aimed to determine the frequency of PS and its associated factors through ultrasonography in people admitted to the hospital in a multicenter prospective study across Turkey.
- Our study showed that the frequency of PS in Turkey is 68.9%, and it is related to age, body mass index, hepatosteatosis, MS (and it’s components). Furthermore, there was a relationship between PS, both pancreatic stiffness and pancreatic exocrine functions.

## Introduction

Excessive lipid accumulation in the pancreas is known as pancreatic steatosis (PS), with synonyms such as fatty pancreas and nonalcoholic fatty pancreas disease. While PS has been a frequent finding in daily ultrasonography practices, its clinical significance and importance were not understood until recently, and objective data regarding its frequency remains unavailable. However, ectopic fat accumulation in the liver, known as fatty liver, and furthermore, nonalcoholic fatty liver disease (NAFLD), which develops in a similar way, has long been associated with an increased risk of diabetes mellitus (DM), metabolic syndrome (MS), and cardiovascular disease.^1-4^ Substantial information has been obtained from recent studies on the prevalence of NAFLD in Turkey.^5,6^ Furthermore, previous studies have reported a strong association between NAFLD and fatty pancreas.^7-10^

The initial publication on PS dates back to Ogilvie’s^11^ article in 1933, which reported its association with weight gain. Approximately, 45 years later, in 1978, Olsen’s^12^ postmortem studies showed the relationship between PS and aging, while Stamm^13^ demonstrated its relationship with type 2 DM and severe atherosclerosis. Additionally, recent studies have shown that PS can disrupt the endocrine and exocrine functions of the pancreas and is associated with MS and insulin resistance (IR).^14^ Sezgin et al^14^ demonstrated that PS and an increase in PS stage detected by ultrasonography were associated with MS and its components and hepatic steatosis (HS). Furthermore, they observed that pancreatic tissue stiffness as determined by (2-dimensional) 2D shear wave elastography increased in direct proportion to PS and the severity of steatosis.^14^ It has been suggested that PS may lead to acute pancreatitis, chronic pancreatitis, or pancreatic cancer.^15-18^ Pancreatic steatosis has been associated with increased mortality in pancreatic cancer, with tumor dissemination, and the formation of pancreatic fistula after pancreatic surgery.^19,20^

Data on the frequency of PS, which carries important clinical implications, is quite limited. Studies, mostly originating from Asian countries, report an average PS frequency of 33%.^21^ However, there is currently no data regarding its frequency in Turkey. Pancreatic steatosis can be easily identified by imaging methods such as transabdominal ultrasonography (TAU), endoscopic ultrasonography (EUS), computed tomography, and magnetic resonance imaging (MRI). In particular, TAU is a safe, rapid, inexpensive, and most widely available imaging tool, and its effectiveness has been demonstrated in comprehensive epidemiological studies.^8,9,22-27^

Based on these considerations, as the Turkish Pancreas Study Group, we aimed to assess the prevalence of PS detected by TAU and identify the factors associated with PS and its severity in a nationwide study in Turkey.

## Materials and Methods

### Study Population

The study was an observational, cross-sectional, prospective, nationwide trial of PS conducted by the “Pancreas Study Group” of Turkish Gastroenterology Association. It took place between May 5, 2021, and June 6, 2021 (data collection period) conducted by gastroenterology clinics associated with the “Pancreas Study Group” that had agreed to participate in the study. All patients who underwent routine TAU examination during daily outpatient clinic evaluation at the participating reference centers were prospectively enrolled. Patients younger than 18 years, those with known pancreatic disease (acute pancreatitis, chronic pancreatitis, or pancreatic cancer), patients who have undergone pancreatic surgery, and those with concomitant liver diseases (such as cirrhosis, but not steatosis) were excluded from the study. The study protocol was approved by the Ethics Committee of the Mersin University Medical School (date: November 15, 2021; number: 91). Written informed consent was obtained from the patients who agreed to take part in the study.

### Clinical and Biochemical Parameters

Systolic blood pressures (SBP) and diastolic blood pressures (DBPs) of the subjects were measured in the sitting position, and those with SBP >130 mmHg and/or DBP >85 mm Hg were defined as hypertension (HT). Waist circumference (WC) was assessed in the middle portion between the 12th rib margin and the upper part of the iliac crest, during the end of the respiratory expiration. Body mass index [BMI: weight (kg)/height^2^ (m^2^)] was calculated. Following overnight fasting, complete blood count, liver tests [aspartate aminotransferase (AST), alanine aminotransferase (ALT), total bilirubin, alkaline phosphatase (AP), and gamma glutamyl transferase (GGT)], fasting blood glucose (FBG), insulin, glycated hemoglobin A1c (HbA1c), lipid profile [total cholesterol, high-density lipoprotein (HDL) cholesterol, low-density lipoprotein (LDL) cholesterol (LDL cholesterol calculated using the Friedewald formula)], triglyceride (TG), blood urea and creatinine, lactate dehydrogenase (LDH), and erythrocyte sedimentation rate (ESR) were evaluated.

Insulin resistance was assessed using the homeostasis model assessment (HOMA-IR) formula (fasting insulin (mU/mL) × fasting plasma glucose (mg/dL)/405). Patients with a HOMA-IR below 2.5 were considered normal, while those with a HOMA-IR of 2.5 or higher were classified as having IR. Diabetes mellitus was defined according to the American Diabetes Association recommendation.^28^ Metabolic syndrome was diagnosed when 3 or more of the following criteria (according to the National Cholesterol Education Program Adult Treatment Panel-III) were present: abdominal obesity (WC >90 cm in men and >80 cm in women), increased TG concentration (>150 mg/dL), decreased HDL-C (<40 mg/dL in men or <50 mg/dL in women), HT (SBP >130 mm Hg and DBP >85 mm Hg), and high FBG concentration (> 110 mg/dL).^29^ Fecal elastase measurement was performed, if available in that study center, to evaluate pancreatic exocrine function.

### Transabdominal Ultrasonography Examination

Transabdominal ultrasonography was conducted following a 12-hour fasting period by gastroenterologists experienced in TAU who performed more than 500 procedures. Before starting the study, video training sessions were conducted to achieve uniformity in ultrasonographic evaluation and nomenclature. Size, echogenicity, and contours of the head, body, and tail of the pancreas were evaluated. Pancreatic echogenicity was compared with liver echogenicity at the same depth on a longitudinal scan taken near the midline of the abdomen. If increased echogenicity was detected in the liver, comparison was made with the renal cortex. If pancreatic echogenicity was found to be increased compared to the liver or kidney cortex, PS was diagnosed. Since the pancreas cannot be compared with the kidney in the same ultrasound window, the ultrasonographer compared the difference between hepatic and renal echogenicity and between hepatic and pancreatic echogenicity to achieve an objective ultrasound contrast between the pancreas and kidney. Pancreatic echo was then staged according to the intensity of echogenicity. The grading system used to evaluate pancreatic echogenicity was adapted from previous grading systems used by Marks et al,^24^ Worthen and Beabeau,^25^ Lee,^30^ and Sezgin et al^14^ as follows: Grade 0 (normal) indicated that the echogenicity of the pancreas is equal to that of the liver. If liver steatosis was present, the pancreas was compared with kidney or spleen echogenicity. Grade I (mild steatosis) signified a slight increase in echogenicity of the pancreas compared to the liver, with distinct borders of the pancreas and a clear observation of the splenic vein. Grade II (moderate steatosis) was assigned when the echogenicity of the pancreas was definitely higher than that of the liver but lower than the echogenicity of retroperitoneal fat with blurred borders of the pancreas. Grade III (severe steatosis) was assigned when the echogenicity of the pancreas equaled or exceeded that of retroperitoneal fat, rendering the evaluation of the pancreas borders and monitoring the splenic vein impossible and causing a cloud cluster-like pattern in the pancreas.

Hepatic steatosis was determined by evaluating transverse and longitudinal ultrasonic images of the liver, diaphragm, and right kidney. Hepatic steatosis was defined based on specific liver echo characteristics as follows: grade 0 (normal liver echogenicity) indicates that the echogenicity of the liver parenchyma is equal to the echogenicity of the kidney. Grade I (mild steatosis) is characterized by normal visualization of the diaphragm and intrahepatic vascular borders with an increase in liver parenchymal echo. Grade II (moderate steatosis) signifies poor visualization of the diaphragm and intrahepatic vascular boundaries, along with posterior attenuation and increased liver parenchymal echogenicity. Grade III (severe steatosis) is characterized by a significant increase in liver parenchymal echogenicity accompanied by significant posterior attenuation, making it impossible to distinguish intrahepatic vessel walls, and the diaphragm.^31-33^

### Two-Dimensional Shear Wave Elastographic Evaluation of the Pancreas and Liver

Pancreas and liver elastography was carried out on patients at centers with access to transabdominal ultrasonographic shear-wave elastography (SWE) facilities. Patients were asked to hold their breath during the ultrasonographic examination and following successful image stabilization, the pancreas was clearly visualized without obvious motion artifacts. Subsequently, a region of interest (ROI) window measuring 10 × 10 mm was placed on the pancreatic tissue on the ultrasonography monitor, with special attention to avoiding contact with the liver parenchyma, adjacent vessels, or structures of the digestive tract. After positioning the ROI window, a shear wave elastography impulse was triggered. After a short while (about 1-2 seconds), pancreatic stiffness (kPa) was displayed, along with the depth of the ROI placement. Five measurements were taken for each segment of the pancreas, and the median of these 5 measurements was considered the valid value. We used transverse or slightly oblique transverse sections.^34,35^ Liver SWE measurement was taken from the right intercostal space. After the right lobe of the liver was visualized ultrasonographically, the ROI window was placed 1-3 cm below the liver capsule and a SWE impulse was triggered. The median value of 10 measurements (kPa) was considered as the valid measurement.

### Statistical Analysis

Descriptive statistics were used as numbers and percentages for categorical variables, and mean ± SD, or median for continuous variables. Normal distribution was investigated visually (by using histograms) or by analytical methods (Kolmogorov–−Smirnov/Shapiro–Wilk tests). Chi-square analysis was used in the analysis of categorical variables. Student *t*-test was used for continuous variables when the normal distribution condition was met; If the normal distribution condition was not met, Mann–Whitney *U*-test was used for comparison between the groups. For the multivariate analysis, the possible factors identified with univariate analyses were further entered into the logistic regression analysis to determine independent predictors of PS. Hosmer–Lemeshow goodness of fit statistics were used to assess model fit. A 5% type-1 error level was used to infer statistical significance. A *P* value of less than .05 was considered to show a statistically significant result.

The inter-observer agreement within the study group, determining the PS and severity of PS on TAU, was investigated using the Kappa test. After 1 observer from the others (OS) was assumed to be the gold standard, on this discrimination, other each observer was paired to OS, and an individual Kappa test was performed for each pair of observers. The interobserver agreements for the severity of PS on TAU between each indvidual observer and the gold standard observer were interpreted as follows: poor, <0.20; fair, 0.20-0.39; moderate, 0.40-0.59; substantial, 0.60-0.79; and almost perfect, ≥0.80.

## Results

### Basic Clinical Parameters of the Study Population

A total of 1700 volunteers from 14 centers participated in the study. Out of them, 967 (56.9%) were female with a mean age of 48.63 ± 24.03 years and 733 (43.1%) were male with a mean age of 47.25 ± 15.71 years. The mean age of the entire cohort was 48.03 ± 20.86 years. There was no significant difference between the genders with respect to age (*P* = .176). The mean BMI of the cohort was 27.22 ± 5.00 kg/m^2^, with 30.7% being normal, 2% underweight, 42.8% overweight, and 24.4% obese. The frequency of DM within the cohort was 17.2%. The frequencies of HT and MS were 23.9% and 26.4%, respectively.

### Prevalence of Pancreatic and Hepatic Steatosis

**Figure 1.**
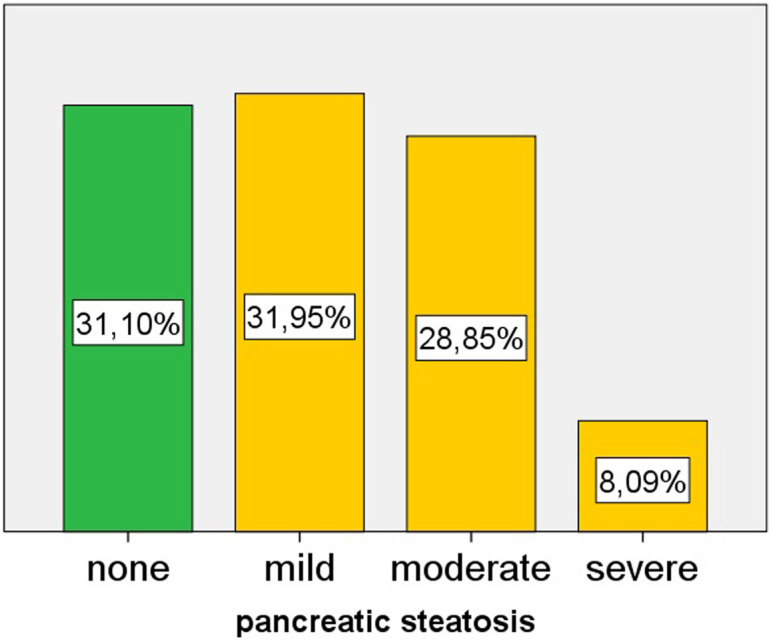
Pancreatic echogenicity of the patients in the study cohort.

**Figure 2.**
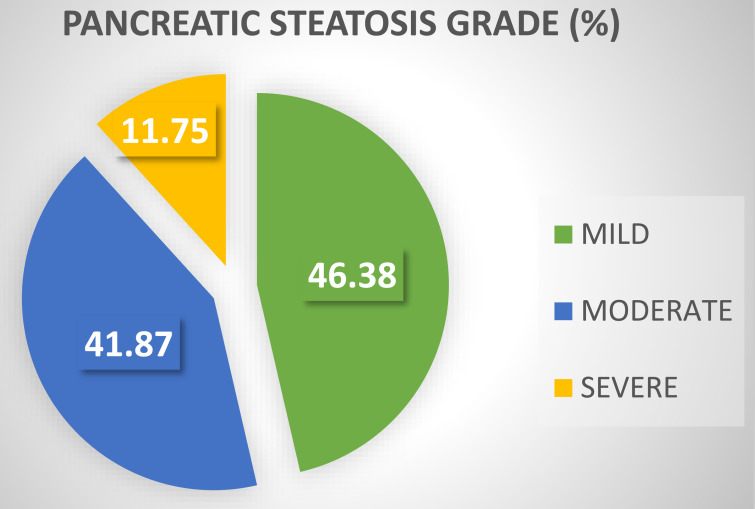
Severity of steatosis in patients with pancreatic steatosis.

Ultrasonography revealed normal pancreatic echogenicity in 31.1% of the cohort with no PS. The remaining 68.9% had PS. Among the patients with PS, 32% had mild PS, 28.8% had moderate PS, and 8.1% had severe PS (Figure 1). Among the cases with PS, 46.4% were categorized as mild, 41.9% as moderate, and 11.7% as severe (Figure 2). The pancreas could be visualized in all of the patients. The interobserver agreement for assessing the severity of PS on US ranged from a maximum of “almost perfect” (kappa: 0.893) to a minimum of “substantial” (kappa: 0.629). Hepatic steatosis was observed in 55% of the cohort. The clinical, laboratory, and ultrasonographic findings of the patients with and without PS are presented in Table 1.

Patients with PS were found to be significantly older and have significantly higher BMI, WC, and SBP values, as well as higher AST, ALT, ALP, GGT, FBG, total cholesterol, TG, LDL, and insulin levels compared to patients without PS. Additionally, patients with PS had significantly higher frequencies of IR, MS, DM, HT, and HS, and lower levels of fecal elastase compared to those without PS.

### Risk Factors for the Occurrence of Pancreatic Steatosis

Multivariate logistic regression was performed to assess the independent risk factors for the occurrence of PS (Table 2). Increased age [odds ratio (OR): 1.02], BMI (OR: 1.089), and the presence of HS (OR: 9.472) were found to be independent risk factors for the occurrence of PS.

### Correlation of the Severity of Pancreatic Steatosis with Clinical, Laboratory, and Ultrasonographic Parameters

In patients with PS, the severity of steatosis showed positive correlations with age, BMI, waist and hip circumference, as well as values for SBP, and serum levels of AST, ALT, ALP, GGT, LDH, FBG, total cholesterol, TG, LDL, and liver and pancreas SWE values. The severity of steatosis was also positively correlated with the frequency of IR, HS, MS, DM, and HT. Additionally, steatosis was negatively correlated with HDL and fecal elastase levels (Table 3).

### Relationship Between Pancreatic Steatosis and Metabolic Syndrome

**Figure 3.**
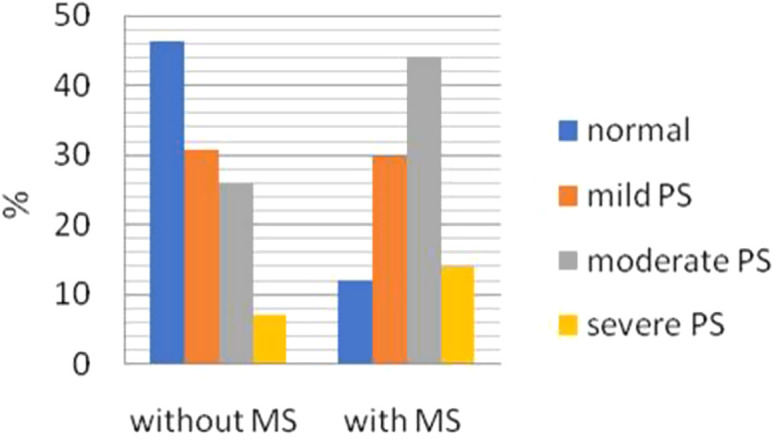
Relationship between metabolic syndrome and severity of pancreatic steatosis.

Metabolic Steatosis was observed in 32.6% of the patients with PS and in 10.1% of the patients without PS. As the degree of PS increased, the frequency of MS increased (Table 3). Among patients without MS, 64% had PS with 30.8% classified as mild, 25.8% as moderate, and 7% as severe PS. However, among those with MS, 88% had PS, with 29.7% being mild, 44.1% moderate, and 14.1% severe PS (Figure 3). Moderate to severe PS was more frequent in patients with MS (*P* = .000). Positive predictive value of PS for assessing the presence of MS was found to be 88%. Patients with both PS and MS had higher values for BMI, WC, hip circumference, SBP, AST, ALT, ALP, WBC, cholesterol, TG, and LDL, had a higher frequency of DM and HT, and lower levels of HDL compared to those with either 1 or none of them (*P *= .000).

### Pancreatic Steatosis and Pancreatic Exocrine Functions

**Figure 4.**
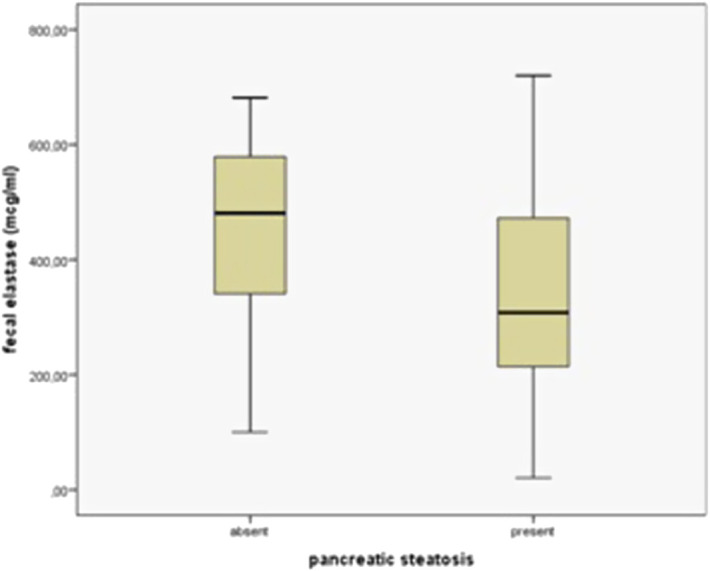
Fecal elastase levels in patients with and without pancreatic steatosis.

**Figure 5.**
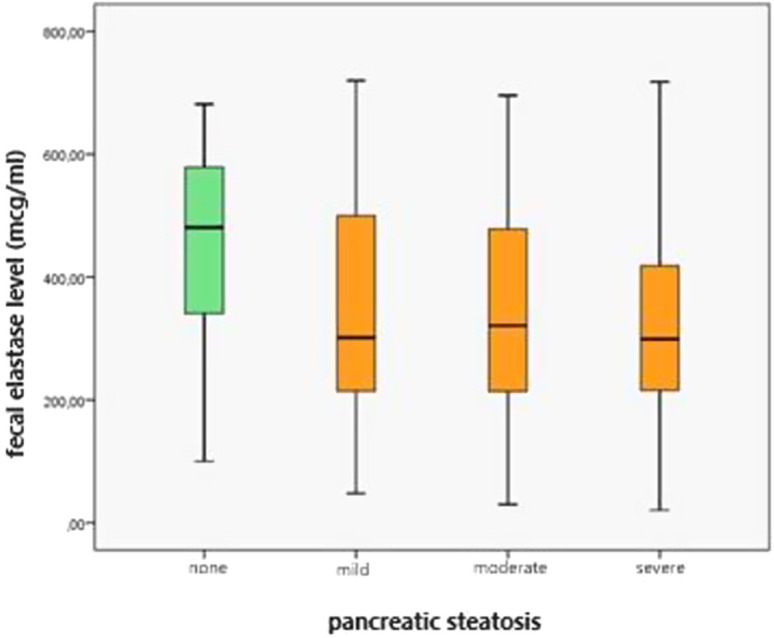
Fecal elastase levels according to the severity of pancreatic steatosis.

Fecal elastase levels were measured in 127 patients. A significant relationship was found between the presence of PS and mean fecal elastase levels (*P *= .001). Fecal elastase levels were 445.30 ± 162.14 µg/mL in patients without PS and 338.18 ± 165.18 µg/mL in patients with PS (Table 1) (Figure 4). Fecal elastase levels were 341.25 ± 178.78 µg/mL in patients with mild PS, 343.04 ± 154.65 µg/mL in moderate PS, and 312.83 ± 154.65 µg/mL in severe PS (*P *= .011) (Table 3) (Figure 5). The rate of fecal elastase levels below 200 µg/mL was 13% in those without PS and 22% in those with PS, and no significant difference was found between the 2 groups (*P * = .337). There was no significant difference between these 2 groups (fecal elastase <200 µg/mL and >200 µg/mL) with respect to all other parameters. The prevalence of EPI was 27.5% in the entire cohort.

### Evaluation of the Pancreas and Liver Stiffness Using Shear Wave Elastography

**Figure 6.**
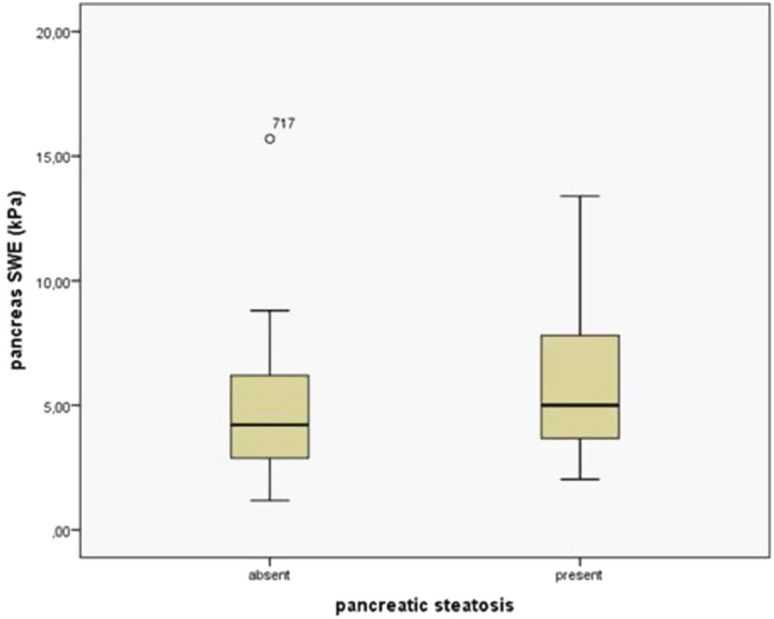
Pancreatic shear-wave elastography values in patients with and without pancreatic steatosis.

**Figure 7.**
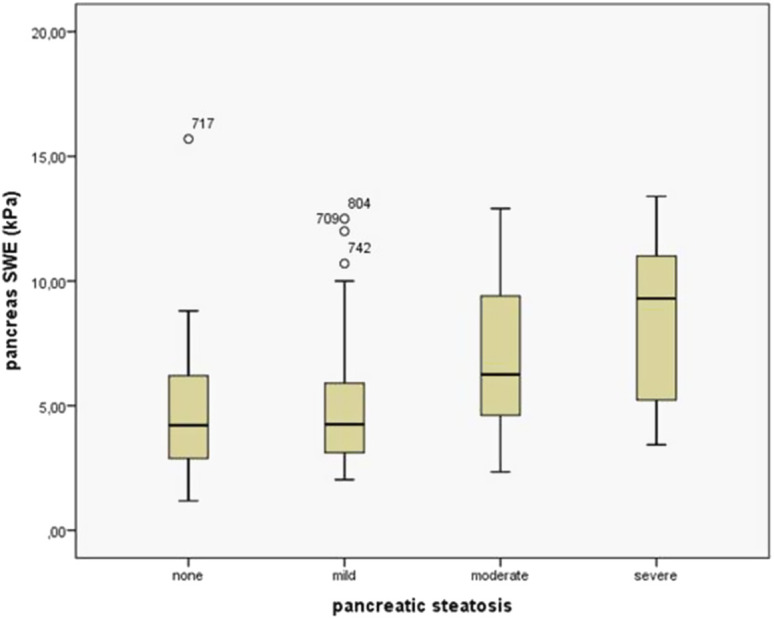
Pancreatic shear-wave elastography values according to the severity of pancreatic steatosis.

A total of 294 patients underwent pancreatic and liver SWE evaluation. The mean pancreas SWE value for the entire cohort was 5.56 ± 2.72 kPa. Patients with PS had significantly higher stiffness compared to those without PS (5.91 ± 2.79 kPa vs. 4.65 ± 2.31 kPa respectively, *P *= .000) (Figure 6). As the severity of PS increased, pancreatic SWE values also increased (*P *= .000) (Figure 7). In the mild PS group, the SWE value was 4.85 ± 2.16 kPa, in the moderate PS group it was 6.97 ± 2.77 kPa, and in the severe PS was 8.49 ± 3.43 kPa. The mean liver SWE value for the entire cohort was 5.62 ± 1.93 kPa, and there is no difference between liver SWE values in patients with or without PS (5.71 ± 1.57 kPa vs. 5. respectively, *P *= .140).

### Relationship Between Hepatic and Pancreatic Steatosis

Among the cohort, 757 patients had HS (55.5%). The severity of HS was mild in 56%, moderate in 31%, and severe in 13%. When the patients were categorized according to the presence of HS and PS, a significant difference was detected between the groups in terms of the distribution of HS and PS (*P * = .000). While the rate of HS in those without PS was 21.5%, the frequency of HS in those with PS was 68.5% (Table 1). Most of the patients without PS did not have HS (78.5%). Of those without HS, 47% had PS. However, 87.5% of patients with HS had PS. The positive predictive value of HS for the presence of PS was calculated as 87.58%. The positive predictive value of PS for the presence of HS was calculated as 68.55%. There was a relationship between the severity of PS and the presence of HS. The HS rate was 57% in mild PS patients, 74% in moderate PS patients, and 90% in severe PS patients (*P *= .000) (Table 3). BMI, WC, SBP, DBP, liver SWE, pancreas SWE values, FBG, ALT, GGT, cholesterol, TG, insulin, HbA1c levels were higher in patients with combined HS and PS compared to those with isolated PS, isolated HS, or neither. Additionally, serum levels of HDL were lower and the frequencies of IR and MS were higher in the combined group.

### Prevalence and Features of Lean Pancreatic Steatosis

There were 201 patients with lean PS, representing 11.8% of the entire cohort. Among the patients with PS, 17% were lean. The comparison of clinical, laboratory, and sonographic findings of the patients with lean and non-lean PS is presented in Table 4. Younger age and a predominantly female gender were observed in patients with lean PS compared to the non-lean patients with PS. Also, in the lean PS group, SBP and DBP values, as well as the levels of FBG, AST, ALT, ALP, GGT, total cholesterol, TG, insulin, and HbA1c, were lower, while HDL levels were higher compared to the non-lean PS group. Moreover, patients with lean PS had lower rates of HS, DM, HT, and MS compared to the non-lean patients with PS. When the patients with lean-PS were compared to lean patients without PS, lean patients without PS were younger and had lower values of BMI, WC, SBP, and DBP, as well as lower levels of FBG, ALT, ALP, GGT, cholesterol, TG, LDL, and insulin levels compared to patients with lean PS. Additionally, lean patients without PS had lower frequencies of IR, DM, HT, MS, and HS. Fecal elastase levels were higher, and pancreas and liver SWE values were lower (Table 4).

## Discussion

This is the first large-scale study investigating the frequency and related factors of PS in Turkey. Our results showed that the frequency of PS was 70% on average. There was a significant correlation between PS and age, BMI, WC, SBP, FBG, serum AST, ALT, ALP, GGT, total cholesterol, TG, LDL, and serum insulin levels, as well as the frequencies of IR, MS, DM, HT, and HS. Patients with PS had lower HDL levels. In PS, the frequency of DM, MS, and HS increased by 3 times, and the frequency of HT increased by 2.3 times. Pancreatic SWE values increased while fecal elastase levels decreased.

In a previous study conducted by Sezgin et al^14^ in Turkey, similar results were observed in the PS group detected by TAU. Patients with PS were older, overweight, with increased WC. They also had higher SBP and a significant increase in the frequency of HT from 14.7% to 1.7%. FBG, serum cholesterol, TG, AP, GGT, AST, ALT levels were also higher in patients with PS. Furthermore, the prevalence of patients with IR (24.9% vs. 8.8%) and those with HbA1c >5.7 (56.8% vs. 21.8%) were greater. The incidence of DM (13.2% vs. 3.5%) and MS (20.5% vs. 3.5%) significantly increased in the PS group. There was a strong relationship between the number of MS criteria and the presence of PS. Patients with PS had a higher OR (5.49) for MS.^14^ The results were similar to those of Wang et al^22^ and Wu and Wang,^36^ indicating a significant relationship between PS and age, obesity, increased SBP, hyperglycemia, and dyslipidemia. The study revealed that patients who had ultrasonographic PS were at an increased risk of MS.

Pancreatic steatosis studies are still in their infancy, and the possible reason why PS entered clinical practice very late is the “myth” that the pancreas cannot be adequately evaluated with TAU in pancreatic imaging and its overlooked value. With a similar fatty condition, the liver has long been the focus of attention and has taken its place in clinical practice. In our opinion, the most important difference in this is that TAU is a very effective and easily applied, safe method in liver imaging and detection of steatosis.^31-33^ Although histopathological evaluation is definitive evidence for the presence of fat infiltrations, and its grade, it requires a biopsy. However, biopsy of the pancreas is an invasive procedure, and not always possible in daily practice. Even if a tissue sample is taken, there may also be grading errors due to the uneven distribution of pancreatic fat.^37^ Therefore, imaging is essential in diagnosis and staging of fatty infiltration of the pancreas. Transabdominal ultrasonography is very effective in pancreas imaging.^14,24-27,38^ Rosenblatt et al^10^ reported a 92% adequacy rate in his ultrasonography study. Pancreatic ultrasonography has a high specificity similar to MRI.^39^ The pancreas can be evaluated with great accuracy and efficiency with TAU, especially for determining its steatosis.^9,14,22,23,40,41^ As our study was multicenter, we aimed to ensure that all researchers were in harmony in the diagnosis and staging therefore conducted a training and practice program before the study. The interobserver agreement for assessing the severity of PS on TAU ranged from a maximum of “almost perfect” (kappa: 0.893) to a minimum of “substantial” (kappa: 0.629). The researchers had good consistency in classifying the pancreas echogenicity.

In 1980, Marks et al^24^ proposed for the first time that ultrasonographic hyperechoic pancreas grade may be related to the amount of intrapancreatic fat. The next study grading the ultrasonographic fatty pancreas and evaluating the association between the severity and clinical and laboratory findings was conducted in a 2009 study by Lee et al^30^ and showed that HOMA-IR, visceral fat, TG, and ALT levels tended to increase with the degree of fat deposition in the pancreas on ultrasonography. Later on, Rosenblatt et al^10^ demonstrated the correlation between ultrasonographic PS grade and NASH. Recently, Sezgin et al^14^ reported that the grade of PS identified by an increase in ultrasonographic pancreatic echogenicity was associated with age, BMI, WC, SBP, serum TG and ALP levels, presence of HbA1c >5.7, IR, fatty liver MS, and its components. Moreover, pancreatic SWE was positively correlated with PS grade, liver fat, MS, and the number of MS criteria.^14^ In the current study, an increase in PS degree was associated with increased age, higher BMI, waist and hip circumference and SBP values, higher FBG, serum AST, ALT, AF, GGT, LDH, total cholesterol, TG, LDL, insulin levels, decreased HDL levels, and increased IR. It was accompanied by an increase in the frequency of HS, MS, DM, HT, and an increase in liver and pancreas SWE values. Fecal elastase levels decreased as the PS degree was increased.

Data on the prevalence of PS in the population is limited, with most studies conducted in Asian countries. In the studies listed chronologically in Table 5,^9,21-23,30,36,37,40,^ ^42-55^ the characteristics and numbers of the examined groups were heterogeneous, leading to highly variable prevalence data. The significant variation in the prevalence rates may be due to the differences in the examined groups, screening methods, the retrospective nature of some studies, and nonstandard definitions. In addition, it has been suggested that ethnic differences may also play a role in the development of PS. For example, in a study conducted in the United States, obese Hispanics accumulated a higher pancreatic TG content than obese African Americans.^56^ Hispanics and Caucasians had a higher risk of developing pancreatic fat infiltration compared to African Americans.^56,57^

Factors associated with PS across the studies include overweight, obesity, DM, MS, NAFLD, and HT.^9,14,21,30,37,48^ In our study group, overweight and obesity rates were 42.8% and 24.4%, respectively. Studies on obesity in Turkey showed that overweight and obesity are common and their prevalence has been increasing over time. Population-based studies on this topic in Turkey were conducted in 2001 and 2012.^58,59^ The latest one showed that 32% of the general population in Turkey were obese. The prevalence of obesity has increased by 44% in 12 years. In the new and largest community-based population study, Cappadocia cohort, among the adult subjects over 18 years of age, the mean BMI of the cohort was 29.7 ± 5.67 ± 5.6 kg/m^2^, with 35.2% being overweight and 45.2% obese.^60^ Similarly, all over the world, there has been a significant increase in overweight and obesity. Nearly 70% of the US adult population is either obese or overweight.^61^

Obesity results in visceral fat deposition in non-adipose tissues such as the liver, heart, skeletal muscle, and pancreas.^62-66^ Much emphasis is placed on the coexistence of fatty liver and pancreas^7-10,14,22,23,30,36,40,47^ Singh et al^21^ showed that the highest limit of normal pancreatic fat in healthy persons in MRI studies was 6.2%. Whichever method is used, pancreatic fat accumulation up to 6.2% is considered normal, and fat accumulation above this is considered excessive. The first histopathological study regarding the association of PS with NAFLD was conducted by van Geenen et al^7^ in an autopsy series. They found that half of the patients with PS had fatty liver disease, with a cutoff value for total pancreatic fat amount >15% associated with NAFLD. When the amount of pancreatic fat exceeds 15%, the liver begins to become steatotic. The important point here is that the pancreas becomes fatty before the liver, and the liver accumulates fat after the pancreas. An ultrasonographic study conducted by Lee et al^30^ showed that 68% of cases with PS also had fatty liver. Of those with fatty liver, 97% also had fatty pancreas. In PS, the positive predictive value for the presence of fatty liver was around 70%, but in the normal pancreas, the negative predictive value for fatty liver was 96%. HS has been identified as the strongest predictor for PS, with a high odds ratio of 14.^30^ In Sezgin et al’s^14^ previous study, the prevalence of NAFLD was significantly higher in patients with PS than in those without PS (73.5% vs. 29.8%). These results were similar to the rate of NAFLD in those with PS reported by Wang [with and without PS: 67% vs. 35%].^22^ Positive predictive value (PPV) and negative predictive value (NPV) of PS for the presence of NAFLD were 78.82% and 77.33%, respectively. Rosenblatt et al^10^ showed that increased severity of PS on ultrasonography (grade II or III echogenicity) was strongly associated with the presence of NASH and advanced fibrosis in the liver (OR 5.37). The HS rate in our study group was 55%. This result was quite consistent with the 60% rate found in the previous Cappadocia Cohort study,^5^ which was important in terms of Turkish data. While the rate of HS was 21.5% in those without PS, the frequency of HS in those with PS was 68.5%. Most of those without PS did not have HS (78.5%). While 87.5% of those with HS had PS, the HS rate was 57% in those with mild PS, 74% in moderate PS, and 90% in severe PS (*P *= .000). While the positive predictive value of HS for the presence of PS was 87.58%, the positive predictive value of PS for the presence of HS was determined as 68.55%. In multivariate analysis (logistic regression analysis), the most important predictor for the presence of PS was found to be HS (relative risk [RR]: 9.472) (Table 2). Body mass index, WC, SBP, DBP, liver SWE, pancreas SWE values, FBG, ALT, GGT, cholesterol, TG, insulin, and HbA1c levels were higher in patients with combined HS and PS compared to other groups with isolated PS, isolated HS, or neither. High-density lipoprotein blood levels were lower and the frequency of IR and MS were higher in patients with PS.

Both HS and PS are risk factors for MS and cardiovascular disease. Due to their obvious association with HT, DM, and HL, they may serve as important indicators for CV risk and the development of these diseases.^51,67^

Human and animal studies have demonstrated that intrapancreatic lipid accumulation leads to oxidative stress and causes cytokine release, such as adiponectin, leptin, TNF-alpha, interleukin-6. This inflammation can lead to ß-cell dysfunction and loss, resulting in DM.^22,67,68^ In a study, patients with PS had an increased risk of diabetes compared with those without PS (12.6% vs. 5.2%), and DM was independently associated with pancreatic fatty infiltration.^22^ In our study, the prevalence of DM was 3 times higher in patients with PS than in those without (21% vs. 7.5%, respectively, *P *= .000). It is possible that PS may promote the development of DM independently of adiposity and other cardiometabolic risk factors.^43^ Some studies have shown that subjects with type 2 DM had higher pancreatic fat content than that of non-diabetics in MR spectroscopy or MRI measurements.^45,48,69^ Also, in healthy individuals, pancreas fat levels have been shown to be negatively correlated with the endocrine functions of the pancreas.^70^ In our study, the frequency of DM increased in correlation with the degree of PS (15% in mild PS, 25% in moderate PS, 25% in severe PS, *P *= .000). The relationship between PS, glycemic progression, and the development of denovo DM has been clearly demonstrated.^40-42,46^ These studies also showed that the risk of glycemic progression significantly increased with the severity of PS, independent of NAFLD or other clinical parameters (HR: 2.718 in mild PS, 6.365 in moderate PS, and 8.984 in severe HP).^37,46^

The frequency of HT in the entire study group was 23.9%. The rate was 12.6% in those without PS and 29.0% in those with PS (*P *= .000). The mean SBP was higher in those with PS than in those without PS. DBP was similar in the 2 groups. The frequency of HT also increased in relation to the degree of PS. Choi et al^43^ found that the fatty pancreas was associated with systolic rather than diastolic HT. In a recent study, the prevalence of HT in our country was found to be 24% in individuals over the age of 15.^71^

Total cholesterol and TG levels were significantly higher in patients with PS compared to those without PS. HDL and LDL levels were correlated with the presence and severity of PS (Table 1 and 3). Elevated TG, HT, MS, and their components are all significant factors of PS development.

Mean serum insulin levels and HOMA-IR score were significantly higher in patients with PS compared to those without PS. The prevalence of MS was also significantly higher in patients with PS. A significant relationship was found between MS and severity of PS. As the degree of PS increased, serum insulin levels, the frequency of IR and MS increased. Moderate and severe PS were more common in individuals with MS. The positive predictive value of PS for the presence of MS was determined as 88%. MS was more common if PS was present. Patients with combined PS and MS had higher BMI, WC, hip circumference, SBP, serum AST, ALT, AF, WBC, cholesterol, TG, LDL levels, and more frequent DM and HT, but lower serum HDL levels compared to patients with isolated MS, isolated PS, and neither. In the study conducted by Ye Bi et al in 2018 on the MS-PS relationship, PS was strongly associated with the occurrence of MS and its components such as HT and DM in the 5-year follow-up.^72,73^ Obesity, HS, and increasing number of MS parameters were predictive of PS. This supports the hypothesis that fatty pancreas is a manifestation of MS. Thereby, PS detected in the course of time can help early detection of MS and reduce cardiovascular risk and improve patient prognosis. The high rate of PS in Turkey can be attributed to the high prevalence of overweight, obesity, along with concomitant presence of DM, HT, and HL. A sedentary life may also play a role.

Pancreatic steatosis can disrupt the endocrine as well as exocrine functions of the pancreas. In our study, we observed a significant relationship between the presence of PS and average fecal elastase levels. Fecal elastase levels were lower in patients with PS compared to those without PS. As the degree of PS increased, the fecal elastase levels decreased. The EPI rate (fecal elastase <200 µg/mL) of the entire cohort was 27.5%, and there was no difference between the patients with and without PS. Given the toxic effect of fat on acinar cells, EPI could occur in the evolution of PS. Two studies to date have evaluated EPI in PS. Tahtacı et al^74^ reported that fecal elastase levels were significantly lower, and the EPI rate was higher in patients with PS (35.5% vs. 12% *P *= .042). Boğa et al^75^ found that pancreatic fat content detected by MRI-estimated proton density fat fraction (PDFF) was significantly higher in patients with EPI compared to those without EPI.

In the current study, pancreatic stiffness was assessed by ultrasonographic 2D SWE. A significant relationship was found between PS and pancreatic SWE. As the severity of PS increased, pancreatic stiffness also increased. In the initial study conducted by Sezgin et al^14^ to evaluate pancreatic stiffness in PS, it was shown that pancreatic stiffness increased in those with PS. There was no difference in pancreatic stiffness according to the measurement location within the pancreas. In males, pancreatic stiffness was highly increased in correlation with BMI, WC, IR, serum TG level, and the severity of PS. The presence of HS and MS significantly increased the pancreatic SWM value. As the number of MS criteria increased, the pancreatic SWM value increased significantly. Patients who had a greater pancreatic SWM value were more likely to have MS. Pathophysiological studies are needed to explain the relationship between PS and pancreatic stiffness.

While overweight and obesity are the most important risk factors associated with the presence of PS, a group of patients with normal BMI also had PS. In our study cohort, 32.7% of patients were categorized as lean. Among the lean patients, 46% had PS, while 79% of the non-lean patients had PS. Lean PS constituted 11.8% of the entire cohort. Among all patients with PS, 17% were categorized as lean. The lean HS rate was 13.4%. Singh stated in his article that BMI and WC were not always related to PS. This suggests that there are different phenotypes among patients with PS. Patients with a lower BMI and smaller WC are still at risk of developing PS. In his autopsy study on humans in 1933, Ogilvie^11^ observed that lean individuals had 9% pancreatic fat and obese individuals had 17% pancreatic fat. In other words, there is a risk of PS even if the BMI is normal or low. In our study, the lean PS group was younger, predominantly female and had lower SBP, DBP, FBG, AST, ALT, AF, GGT, total cholesterol, TG, insulin, and HbA1c levels compared to the non-lean patients. Additionally, HDL levels were higher and the frequencies of HS, DM, HT, and MS were lower in the lean group with PS. Lean patients without PS were younger and had lower BMI, WC, SBP, and DBP values, as well as FBG, ALT, AF, GGT, cholesterol, TG, LDL, and insulin levels compared to those with lean-PS and non-lean patients with PS. The frequencies of IR, DM, HT, MS, and HS were lower in the lean patients without PS. Furthermore, lean patients without PS had higher fecal elastase levels and lower pancreatic and liver SWE values. With these features, the lean-PS group may exhibit a progressive change over time and represent individuals at risk of developing MS in the future. We think that long-term follow-up of this population may answer this question.

Our study had several limitations. Interobserver variability as well as variability of US measurements with different US devices might have affected the overall outcomes. On the other hand, the study had a large sample size, multicentric, and evaluated all factors that may affect PS. Moreover, the study used one of the best noninvasive methods for evaluating fat in the liver and pancreas, which has been proven in previous studies to be a reliable, reproducible, and noninvasive screening tool for fatty pancreas.

In conclusion, this multicenter study revealed a PS frequency of almost of 70%. Pancreatic steatosis was associated with MS and its components and the presence of HS and increasing age. Pancreatic stiffness increased in the presence of PS and was correlated with the PS stage. There was a negative relationship between PS and fecal elastase levels. To date, the identification of ectopic fat accumulation in the pancreas does not routinely figure into clinical decision-making and is rarely discussed in current clinical practice. The results of this study support the hypothesis that PS is a part of the MS and is progressive over time.

## Figures and Tables

**Table 1.** Comparison of the Clinical, Laboratory, and Ultrasonographic Findings of the Patients With and Without Pancreatic Steatosis

	Pancreatic Steatosis Absent	Pancreatic Steatosis Present	*P*
Age (years)	41.39 ± 16.12	50.65 ± 14.58	<.0001
Gender (Female/Male)	308/203	617/514	.77
BMI (kg/m^2^)	24.38 ± 3.98	28.40 ± 4.85	<.0001
Waist circumference (cm)	82.02 ± 12.92	96.29 ± 14.37	.009
SBP (mm Hg)	114.84 ± 12.99	121.83 ± 16.68	<.0001
DBP (mm Hg)	76.84 ± 11.63	77.78 ± 11.11	.218
FBG (mg/dL)	97.93 ± 20.98	109.63 ± 34.99	<.0001
AST (U/L)	21.94 ± 15.04	25.24 ± 17.77	<.0001
ALT(U/L)	21.63 ± 19.22	29.10 ± 24.52	<.0001
ALP (U/L)	71.41 ± 37.10	79.45 ± 46.06	.001
GGT (U/L)	24.63 ± 21.54	32.36 ± 27.50	<.0001
LDH (U/L)	180.84 ± 50.35	188.24 ± 68.26	.062
CRP (mg/L)	4.96 ± 4.31	5.26 ± 4.01	.388
WBC (K/mm^3^)	7.19 ± 2.43	8.03 ± 2.35	.458
ESR (mm/hour)	14.87 ± 13.65	15.67 ± 13.81	.337
Total cholesterol (mg/dL)	189.63 ± 52.68	206.69 ± 49.39	<.0001
Triglyceride (mg/dL)	106.28 ± 56.40	146.45 ± 87.19	<.0001
HDL (mg/dL)	53.23 ± 14.04	51.16 ± 15.48	.017
LDL (mg/dL)	119.35 ± 83.03	132.98 ± 74.18	.002
HbA1c (mmol/mol)	4.67 ± 2.81	4.72 ± 2.71	.788
Insulin (µU/L)	9.22 ± 6.50	11.62 ± 7.82	<.0001
HOMA-IR	4.32 ± 3.49	5.30 ± 3.86	<.0001
MS frequency (%)	10.1%	32.6%	<.0001
DM frequency (%)	7.5%	21.0%	<.0001
HT frequency (%)	12.6%	29.0%	<.0001
Fecal elastase (µg/mL)	445.31 ± 162.15	338.18 ± 165.18	.001
Hepatosteatosis frequency (%)	21%	68%	<.0001

AST, aspartate aminotransferase, ALP, alkaline phosphatase; ALT, alanine aminotransferase, BMI, body mass index; CRP, c-reactice protein; DBP, diastolic blood pressure; DM, diabetes mellitus; ESR, erythrocyte sedimentation rate; FBG, fasting blood glucose; GGT, gamma glutamyl transferase; HbA1c, glycated hemoglobin A1c; HDL, high-density lipoprotein; HOMA-IR, homeostasis model assessment insulin resistance; HT, hypertension; LDH, lactate dehydrogenase; LDL, low-density lipoprotein; MS, metabolic syndrome; SBP, systolic blood pressure; WBC, white blood cells.

**Table 2.** Multivariate Logistic Regression Analysis to Identify the Independent Risk Factors for the Occurrence of Pancreatic Steatosis

		*B*	SE	Wald	df	Sig.	Exp (B)	95% CI for Exp (B)	
								Lower	Upper
Step 1a	Age	0.02	0.007	7.778	1	0.005	1.02	1.006	1.035
	BMI	0.085	0.026	10.338	1	0.001	1.089	1.034	1.147
	SBP	0.012	0.008	2.016	1	0.156	1.012	0.996	1.028
	MS	−0.05	0.318	0.025	1	0.876	0.951	0.51	1.774
	DM	0.69	0.393	3.079	1	0.079	1.994	0.922	4.312
	HS	2.248	0.209	115.629	1	0.001	9.472	6.287	14.27
	Constant	−3.841	1.163	10.9	1	0.001	0.021	3	3

BMI, body mass index; DM, diabetes mellitus; HS, hepatosteatosis; MS, metabolic syndrome; SBP, systolic blood pressure.

**Table 3.** Clinical, Laboratory, and Ultrasonographic Parameters of the Patients with Respect to the Severity of Pancreatic Steatosis

	Normal	Mild PS	Moderate PS	Severe PS	*P*
Age (years)	41.39 ± 16.13	47.55 ± 14.08	53.10 ± 15.03	53.92 ± 13.02	<.0001
BMI (kg/m^2^)	24.38 ± 3.98	27.24 ± 4.38	29.20 ± 5.16	30.32 ± 4.42	<.0001
Waist circumference (cm)	82.03 ± 12.93	92.06 ± 13.21	100.27 ± 13.68	101.03 ± 16.29	<.0001
Hip circumference (cm)	97.08 ± 54.08	103.33 ± 12.25	106.37 ± 14.78	110.34 ± 13.68	.001
SBP (mm Hg)	114.84 ± 13.00	120.41 ± 17.73	124.27 ± 15.65	121.94 ± 13.77	<.0001
DBP (mm Hg)	76.85 ± 11.63	77.17 ± 11.88	78.69 ± 10.34	76.86 ± 11.08	.321
FBG (mg/dL)	97.93 ± 20.98	104.67 ± 31.01	114.71 ± 38.86	111.59 ± 33.11	<.0001
AST (U/L)	20.91 ± 9.66	23.04 ± 10.70	24.08 ± 9.50	24.28 ± 13.54	<.0001
ALT (U/L)	20.79 ± 15.94	25.40 ± 18.17	29.56 ± 23.81	34.03 ± 22.57	<.0001
ALP (U/L)	71.41 ± 37.11	77.52 ± 52.57	82.06 ± 39.85	79.63 ± 31.31	.007
GGT (U/L)	24.63 ± 21.54	27.37 ± 19.98	33.65 ± 27.11	39.51 ± 28.39	<.0001
LDH (U/L)	180.84 ± 50.36	187.63 ± 52.23	193.05 ± 92.31	175.11 ± 42.58	.044
CRP (mg/dL)	6.70 ± 6.30	6.99 ± 6.42	8.25 ± 7.14	7.62 ± 6.42	.144
WBC (K/mm^3^)	7.19 ± 2.44	8.83 ± 3.93	7.30 ± 2.48	7.43 ± 1.78	.586
ESR (mm/h)	13.30 ± 10.45	14.44 ± 10.62	14.05 ± 10.52	11.31 ± 9.17	.062
Total cholesterol (mg/dL)	189.63 ± 52.69	206.90 ± 48.97	207.63 ± 48.74	202.75 ± 53.31	<.0001
Triglyceride (mg/dL)	102.31 ± 48.25	117.57 ± 49.53	129.73 ± 48.46	137.00 ± 54.47	<.0001
HDL (mg/dL)	53.23 ± 14.05	53.30 ± 15.58	49.57 ± 14.28	48.32 ± 17.84	<.0001
LDL (mg/dL)	119.35 ± 83.04	136.89 ± 99.26	130.41 ± 39.38	126.27 ± 42.34	.008
					Insulin (µU/L)	8.99 ± 6.52	10.18 ± 7.33	11.82 ± 7.48	14.08 ± 8.10	<.0001
HOMA-IR	4.16 ± 1.97	4.63 ± 1.93	5.36 ± 1.69	4.95 ± 2.23						<.0001
					HbA1C (mmol/mol)	5.50 ± 2.21	5.45 ± 2.12	5.57 ± 2.52	5.62 ± 2.12	.941
Fecal elastase (µg/mL)	445.31 ± 162.15	341.25 ± 178.78	343.04 ± 154.65	312.83 ± 154.65						.011
					Hepatosteatosis frequency (%)	21%	57%	74%	90%	<.0001
MS frequency (%)	10.1%	25%	37%	41%	<.0001
DM frequency (%)	7%	15%	25%	25%	<.0001
HT frequency (%)	12%	23%	33%	34%	<.0001

AST, aspartate aminotransferase, ALT, alanine aminotransferase, BMI, body mass index; DBP, diastolic blood pressure; DM, diabetes mellitus; ESR, erythrocyte sedimentation rate; FBG, fasting blood glucose; GGT, gamma glutamyl transferase; HbA1c, glycated hemoglobin A1c; HDL, high-density lipoprotein; HOMA-IR, homeostasis model assessment insulin resistance; HT, hypertension; LDH, lactate dehydrogenase; LDL, low-density lipoprotein; MS, metabolic syndrome; PS, pancreatic steatosis; SBP, systolic blood pressure; WBC, white blood cells.

**Table 4.** Comparison of the Patients with Lean Pancreatic Steatosis, Non-Lean Pancreatic Steatosis, and Lean Patients Without Pancreatic Steatosis in Terms of Clinical, Laboratory, and Ultrasonographic Parameters

Parameter	Lean Non-PS	Lean PS	Non-lean PS	*P*
Age (years)	36.88 ± 14.63	47.21 ± 15.98	51.36 ± 14.16	<.0001
Gender (Male%/Female%)	38.46/61.54	36.89/63.11	47.80/52.20	.002
BMI (kg/m^2^)	21.78 ± 2.55	22.81 ± 2.32	30.00 ± 4.16	<.0001
Waist circumference (cm)	78.20 ± 11.61	82.79 ± 11.40	99.67 ± 13.03	<.0001
Hip circumference (cm)	97.35 ± 9.03	101.00 ± 79.15	106.93 ± 13.75	.013
SBP (mm Hg)	111.60 ± 14.66	114.51 ± 17.88	123.40 ± 16.04	<.0001
DBP (mm Hg)	72.13 ± 9.70	74.24 ± 10.34	78.31 ± 11.49	<.0001
Hepatic SWE (kPa)	4.77 ± 0.84	5.84 ± 2.57	5.68 ± 1.27	.001
Pancreatic SWE (kPa)	3.78 ± 1.66	6.35 ± 2.77	5.77 ± 2.79	<.0001
FBG (mg/dL)	94.39 ± 13.98	103.22 ± 30.73	111.25 ± 35.71	<.0001
AST (U/L)	20.53 ± 11.03	21.66 ± 12.21	25.70 ± 16.81	<.0001
ALT (U/L)	20.57 ± 18.23	24.67 ± 22.48	30.34 ± 24.94	<.0001
ALP (U/L)	69.51 ± 38.12	73.07 ± 36.17	80.86 ± 48.10	.002
GGT (U/L)	21.01 ± 16.99	26.21 ± 24.97	33.95 ± 27.90	<.0001
LDH (U/L)	177.88 ± 51.68	180.74 ± 54.39	189.87 ± 71.06	.055
CRP (mg/dL)	6.37 ± 5.46	7.35 ± 5.59	7.00 ± 5.60	.284
WBC (K/mm^3^)	7.22 ± 2.75	6.93 ± 2.30	8.33 ± 28.51	.620
ESR (mm/hour)	12.81 ± 9.30	13.34 ± 9.46	15.09 ± 10.43	.004
Total Cholesterol (mg/dL)	183.17 ± 50.26	199,0047.10	208.52 ± 49.75	<.0001
Triglyceride (mg/dL)	93.98 ± 47.11	110.19 ± 56.70	145.61 ± 69.63	<.0001
HDL (mg/dL)	54.34 ± 14.28	55.42 ± 15.87	50.03 ± 15.09	<.0001
LDL (mg/dL)	118.06 ± 114.11	131.06 ± 115.51	133.31 ± 58.25	.060
Insulin (µU/L)	7.10 ± 3.87	7.71 ± 4.49	9.99 ± 4.85	<.0001
HOMA-IR	3.73 ± 1.93	4.50 ± 1.59	4.91 ± 1.82	.002
HbA1c (mmol/mol)	3.01 ± 2.26	3.64 ± 2.48	4.03 ± 2.32	<.0001
Fecal elastase (µg/mL)	446.51 ± 158.97	332.56 ± 166.25	339.95 ± 165.45	.015
DM frequency (%)	3.23	11.52	23.97	<.0001
HT frequency (%)	7.06	15.67	33.14	<.0001
MS frequency (%)	5.38	10.84	39.52	<.0001
HS frequency (%)	18.32	57.79	71.48	<.0001

AST, aspartate aminotransferase, ALT, alanine aminotransferase, BMI, body mass index; DBP, diastolic blood pressure; DM, diabetes mellitus; ESR, erythrocyte sedimentation rate; FBG, fasting blood glucose; GGT, gamma glutamyl transferase; HbA1c, glycated hemoglobin A1c; HDL, high-density lipoprotein; HOMA-IR, homeostasis model assessment insulin resistance; HS, hepatosteatosis; HT, hypertension; LDH, lactate dehydrogenase; LDL, low-density lipoprotein; MS, metabolic syndrome; PS, pancreatic steatosis; SBP, systolic blood pressure; WBC, white blood cells.

**Table 5.** The Prevelance of Pancreatic Steatosis in Previous Series

Country. Author. Reference Number	Year, Population	Number of Participants	Method of Diagnosis	Frequency of PS
Japan. Makino et al^42^3	2005-2006, healthy volunteers	472	TAU	44%
South Korea. Choi et al^43^3	2007, patients referred for EUS examination	284	EUS	38.7%
South Korea . Lee et al ^30^3	2009, undergone a health checkup for obesity	293	TAU	61%
USA. Sepe et al^44^3	2011, patients referred for EUS examination	250	EUS	27.8%
China. Li et al^45^3	2011, healhy male	126	Chemical shift MRI	6.3%
Taiwan. Hung et al^46^3	2005-2011, medical check-up patients	32346	TAU	8.4%
China. Zhou et al^47^3	2013, a health checkup	1190	TAU	30.7%
Indonesia. Lesmana et al^23^3	2013, medical check-up patients	1054	TAU	35%
Taiwan. Wu and Wang^36^3	2013, healthy subjects	557	TAU	12.9%
Taiwan. Wang et al^22^3	2014, a health check-up	8097	TAU	16%
Hong Kong. China. Wong et al^48^3	2014, healthy volunteers	685	MRI and proton-magntic resonance spectrescopy	16.1%
Turkey. Uygun et al^9^3	2014, Hospitalized and outpatient individuals	119	TAU	51% in NASH patients 14% in healthy people
South Korea. Oh et al^40^3	2008-2014, retrospective. health check-up	1366	TAU	69.9%
China. Weng et al^49^3	2015-2017, medical examination or outpatient visit	4419	TAU	11.5%
Chilea. Berger et al^50^3	2015, retrospective	203	TAU	30%
China. Li et al^37^3	2017, medical check-up	256	TAU	47.3%
China, South Korea, Turkey, Italy. Singh et al^21^3	2017, meta-analysis	12 675	TAU. EUS. CT. MRI	33%
South Korea. An et al ^51^3	2017-2018, retrospective medical check-up	544	TAU	62.8%
China. Wang et al^52^3	2018, the staff (including retirees) of this hospital	1228	TAU	4.3%
China. Weng et al^53^3	2018, medical check-up	4368	TAU	10.8%
Iran. Sotoudehmanesh et al^54^3	2019, patients referred for EUS examination	228	EUS	25.9%
Japan. Okada et al^55^3	2020, medical check-up	919	TAU	46.8%
Turkey. Sezgin et al (Current study)	2022-2023, medical check-up	1700	TAU	68.9%

CT, computed tomography; EUS, endoscopic ultrasonography; MRI, magnetic resonance imaging; PS, pancreatic steatosis; TAU, transabdominal ultrasonography.
